# Precision N-Glycoproteomic Profiling of Murine Peritoneal Macrophages After Different Stimulations

**DOI:** 10.3389/fimmu.2021.722293

**Published:** 2021-08-17

**Authors:** Lujie Yang, Tianqi Gong, Huali Shen, Jiangnan Pei, Lei Zhang, Quanqing Zhang, Yuanyu Huang, Zuojian Hu, Ziyue Pan, Pengyuan Yang, Ling Lin, Hongxiu Yu

**Affiliations:** ^1^Institutes of Biomedical Sciences & Shanghai Stomatological Hospital, Fudan University, Shanghai, China; ^2^Obestetics & Gynecology Hospital, Fudan University, Shanghai, China; ^3^Department of Chemistry and Environmental Toxicology Graduate Program, University of California, Riverside, CA, United States; ^4^Department of Clinical Laboratory, First Affiliated Hospital of Guangxi Medical University, Nanning, China; ^5^Xiamen Cardiovascular Hospital, Xiamen University, Xiamen, China

**Keywords:** macrophage, N-glycosylation, Toll-like receptors, glycoproteomics, inflammatory response

## Abstract

Macrophages are important immune cells that participate in both innate and adaptive immune responses, such as phagocytosis, recognition of molecular patterns, and activation of the immune response. In this study, murine peritoneal macrophages were isolated and then activated by LPS, HSV and VSV. Integrative proteomic and precision N-glycoproteomic profiling were conducted to assess the underlying macrophage activation. We identified a total of 587 glycoproteins, including 1239 glycopeptides, 526 monosaccharide components, and 8326 intact glycopeptides in glycoproteomics, as well as a total of 4496 proteins identified in proteomic analysis. These glycoproteins are widely involved in important biological processes, such as antigen presentation, cytokine production and glycosylation progression. Under the stimulation of the different pathogens, glycoproteins showed a dramatic change. We found that receptors in the Toll-like receptor pathway, such as Tlr2 and CD14, were increased under LPS and HSV stimulation. Glycosylation of those proteins was proven to influence their subcellular locations.

## Introduction

Glycosylation is an important post-translational modification of proteins, and glycoproteins are widely involved in various important cellular biological processes and diseases (1). Different glycans of proteins can result in different immunogenicities and immune functions (2). Current research shows that the glycan of a protein can directly affect its structure and can also maintain the stability of the protein (3). The biosynthesis of N-glycans is a highly orderly process. A high-mannose oligosaccharide precursor is synthesized and transferred to proteins in the endoplasmic reticulum and then processed in the Golgi apparatus (4).

For better insight into the role of glycosylation in biological progress and diseases, glycosylation profiling strategies are necessary for glycobiology studies. Large-scale analysis of glycosylation in organisms includes glycomics (glycan profiling) and glycoproteomics (glycosylation site profiling) (5). Mass spectrometry and lectin arrays are common technologies publicly adopted in glycosylation analysis. Lectin array technology is based on the lectin-glycan affinity (6), which is not suitable for glycoproteomic analysis. Mass spectrum-based high-throughput characterization of glycosylation as well as proteomic analysis has been used for years (7–11). Currently, analysis by liquid chromatography coupled with tandem mass spectrometry (LC-MS/MS) of intact glycopeptides is often the method of choice in site-specific glycoproteomic studies (12). Yang’s group developed a fine-tuned MS/MS acquisition method and pGlyco 2.0 search engine to perform confident intact glycopeptide characterization (13).

Macrophages are immune cells widely distributed throughout the body and are components of innate immunity. These cells participate in pathogen recognition, apoptotic cell clearance, and antigen presentation (14). Macrophages have different activation states in different microenvironments (15) and pathogenic stimulation (16). Glycans are one of the four fundamental macromolecular components of all cells and are highly regulated in immune cells, including macrophages. New technology for glycomic and glycoproteomic analysis has been used in macrophage research. The N-glycome showed a dramatic change during human monocyte-to-macrophage transition (17) and murine macrophage polarization (18). Infection with *Mycobacterium* dramatically altered the N-glycosylation of macrophages (19, 20). Multiple studies have shown that N-glycosylation of macrophages is changed in different microenvironments and infection statuses. There is a lack of studies that performed intact glycopeptide analysis of macrophages under stimulation with different pathogens.

Our research applied high-throughput MS acquisition with the pGlyco2.0 search engine to analyze the glycoprotein variation of mouse peritoneal macrophages under LPS, HSV and VSV stimulation. In this study, we acquired both proteomic and intact N-glycoproteomic data and identified 8326 intact glycopeptides among 587 glycoproteins. Under different stimuli, glycoproteins showed significant changes. Through pathway analysis of the changed glycoproteins, we observed significant enrichment in the Toll-like receptor pathway. CD14, Tlr2, and Tlr7 levels were upregulated in response to stimulation with different pathogens. In addition, we proved that inhibiting glycosylation may affect the expression and localization of these proteins. By mutating glycosylation sites in a plasmid, we confirmed that the mutation of N-glycosylation sites directly affects Tlr2 subcellular localization.

## Material and Methods

### Cells and Reagents

RAW264.7 cells were obtained from the Chinese Academy of Sciences. NIH-3T3 was a gift from Wenbo’s laboratory, Fudan University. DMEM and EDTA trypsin were purchased from HyClone (USA). LPS (lipopolysaccharide) was purchased from Sigma-Aldrich (USA). The HSV-1 and VSV strains were kindly provided by Dr F. Qian, Fudan University, China and Dr FJ. Hou, SIBCB, CAS, China. PNGase F was purchased from NEB (England). Trypsin was purchased from Promega (Madison, USA). A C18 column was purchased from Waters (Massachusetts, USA). HILIC Amphion was purchased from Welch (Shanghai, China). The iTRAQ label kit was purchased from AB SCIEX (Framingham, USA). The RT-RNA kit and qPCR kit were purchased from TaKaRa (Japan). CD14, Tlr2, Icam1, and Cox2 antibodies (rabbit) were purchased from Abcam (Cambridge, England). Antibodies (rabbits) against Tlr7 were purchased from Proteintech (Chicago, USA). Anti-rabbit conjugated with 488 fluorescence was purchased from Invitrogen (Waltham, USA).

### Cell Culture

Mouse peritoneal macrophages, RAW264.7 cells and NIH-3T3 cells were cultured in DMEM (containing 10% FBS and 1% penicillin/streptomycin). To inhibit glycosylation of the cell lines, we incubated these two cell lines with DMEM containing tunicamycin (1 μg/ml), kifunensine (1 μg/ml) and swainsonine (1 μg/ml) for 48 hours. Murine peritoneal macrophages were obtained according to a previous study (21) and cultured in DMEM for at least 2 hours at 37°C to make them adhere to the substrate.

All experiments on mice were performed in accordance with the Guiding Principles for Research Involving Animals and Human Beings and approved by the ethics committee at Fudan University.

### Protein Extraction, Tryptic Digestion, and Sample Preparation

Proteins were extracted from peritoneal macrophages using 4% SDS denaturing buffer (Tris-HCl, pH=7.6). Lysis was performed for 30 minutes. The protein concentration was quantified by a BCA kit (Thermo). Before tryptic digestion, 5 volumes of precooled acetone were added to the protein, and the sample was incubated at -20°C. After centrifugation at 140000 rpm for 40 minutes, the supernatant was removed. The protein was resuspended in 50 mM ABC, and trypsin was added at a concentration of 1:50 (w/w) to the protein in the solution. Proteins were digested for 18 hours at 37°C. A C18 desalting column was used to desalt peptides according to the protocol. Approximately 100 µg of desalted peptides from different groups was labeled with iTRAQ 8-plex reagent according to previous studies (22).

### Glycopeptide Enrichment

Lyophilized peptides were reconstituted with loading buffer (80% acetonitrile, 1% TFA). A 200 µL pipette tip was filled with approximately 20 µg ZIC-HILIC to construct a ZIC-HILIC column. Then, 100 µl of washing buffer (80% acetonitrile, 1% TFA) was added to a ZIC-HILIC column and centrifuged at 10000 rpm for 2 minutes, and the eluate was discarded. The column was washed three times. The peptides were loaded onto the ZIC-HILIC column and centrifuged at 10000 rpm for 2 minutes, and the eluate was discarded. The glycopeptide was eluted from peptide with 200 µL of elution buffer 1 (0.1% TFA), 20 µL of elution buffer 2 (50 mM ABC) and 20 µL of elution buffer 3 (50% acetonitrile).

### LC-MS/MS

In quantitative proteomics, prefractionated peptides were labeled with iTRAQ 8-plex reagent and analyzed by LC-MS/MS. Intact N-glycopeptides were quantified using a label-free method. Details are provided in the **Supporting Information**.

### Release N-Glycan on Protein by PNGase F Enzyme

Approximately 20 µg of protein was diluted to a 9 µl volume, 1 µl of 10× protein denaturant was added, and the mixture was boiled in boiling water for 10 minutes and placed on ice. Then, 2 µl of 10× glycoprotein buffer, 2 µl of 10×NPC, and 7 µL of water were added. Finally, 1 µl of PNGase F enzyme was added. The sample was reacted at 37°C for 18 hours.

### Western Blot

The protein was separated by 10% SDS-PAGE at 30 mA for 2 hours in running buffer and then transferred onto a nitrocellulose membrane at 300 mA for 1.5 hours. Then, the cells were blocked in blocking buffer (5% BSA in TBST) for at least 1 hour. The membrane was incubated with a 1:500 to 1:2000 dilution of primary antibody in blocking buffer overnight. The membrane was washed with TBST 3 times. The membrane was incubated with a 1:5000 dilution of HRP-conjugated anti-rabbit secondary antibody in blocking buffer for 1 hour. The membrane was washed three times. Proteins were detected by chemiluminescence and autoradiography.

### Cell Immunofluorescence Assay

The cells were fixed on glass coverslips with 4% formaldehyde for 10 minutes and washed with PBS for 3 minutes, and this procedure was repeated three times. Cells were blocked for 1 hour in 4% BSA dilution in PBS. After blocking, the cells were incubated in a 1:500 dilution of primary antibody overnight. The coverslips were washed 3 times with PBS. The secondary antibody (anti-rabbit-488, anti-rat-Cy3) was diluted 1:2000 and incubated with the cells for 1 hour. The slides were washed with PBS three times. The coverslips were sealed on a glass slide with DAPI Antifade Solution. The samples were stored at 4°C after mounting on the slides for anti-fluorescence quenching (485 nm, 566 nm detection signal).

### Plasmid Constitution and Transfection

The Tlr2 and Tlr2 (N414Q/N442Q) gene sequences were inserted into the GV141 plasmid. The Tlr2 sequences were chemically synthesized (GeneChem, Shanghai, China), and sequencing analysis is shown in **Supporting Information**. Competent bacteria and plasmids were incubated on ice, and 50 ng reconstituted plasmid was added to 5×105 cells, placed on ice for 20 minutes, placed in a 42°C water bath for 90 seconds, and cooled on ice for 2 minutes. Then, the sample was inoculated on solid medium containing 10 μg/ml ampicillin and cultured overnight. Single colonies were selected and inoculated in 20 ml (10 μg/ml ampicillin) of LB medium. The bacteria were shaken and cultivated overnight. After centrifugation at 4000 rpm for 20 minutes, amplified colonies were obtained, and plasmids were extracted from the bacteria *via* a Plasmid Mini Preparation Kit.

Then, 1.5 ml of serum-free medium was added to each well of a six-well plate and incubated for at least 2 hours. Next, 4.5 μl of Lipo3000 reagent was diluted in 120.5 μl of serum-free medium and incubated for 5 minutes. Three micrograms of plasmid and P3000 to 125 μl were diluted with serum-free medium. Then, the plasmid solution was added to the Lipo3000 dilution, slowly mixed by pipetting, and incubated for 25 minutes. The mixed solution containing the plasmid and Lipo3000 was added dropwise to the six-well plate. After incubation for 6 hours, the medium was changed. Samples were collected after 48 hours of incubation.

### Data Analysis

The intact glycopeptides were identified by the pGlyco2.0 search engine. The intact glycopeptides were quantified by iBAQ. Proteomic data were obtained and quantified by PEAKS. Statistical analysis was performed with R-software. Differential analysis was applied by the limma package (23). A heat map with a clustering tree was generated by the pheatmap package, and hierarchical clustering was performed according to the Euclidean distance measure. Gene Ontology analyses were performed according to the R packages clusterProfiler (24) and the org.Mm.eg.db package. The network diagram was generated by using Cytoscape. Colocalization was analyzed using ImageJ and Fuji software.

## Results

### Quantitative Proteome and N-Glycoproteome in Murine Peritoneal Macrophages Under Different Pathogenic Stimuli

**Figure 1:**
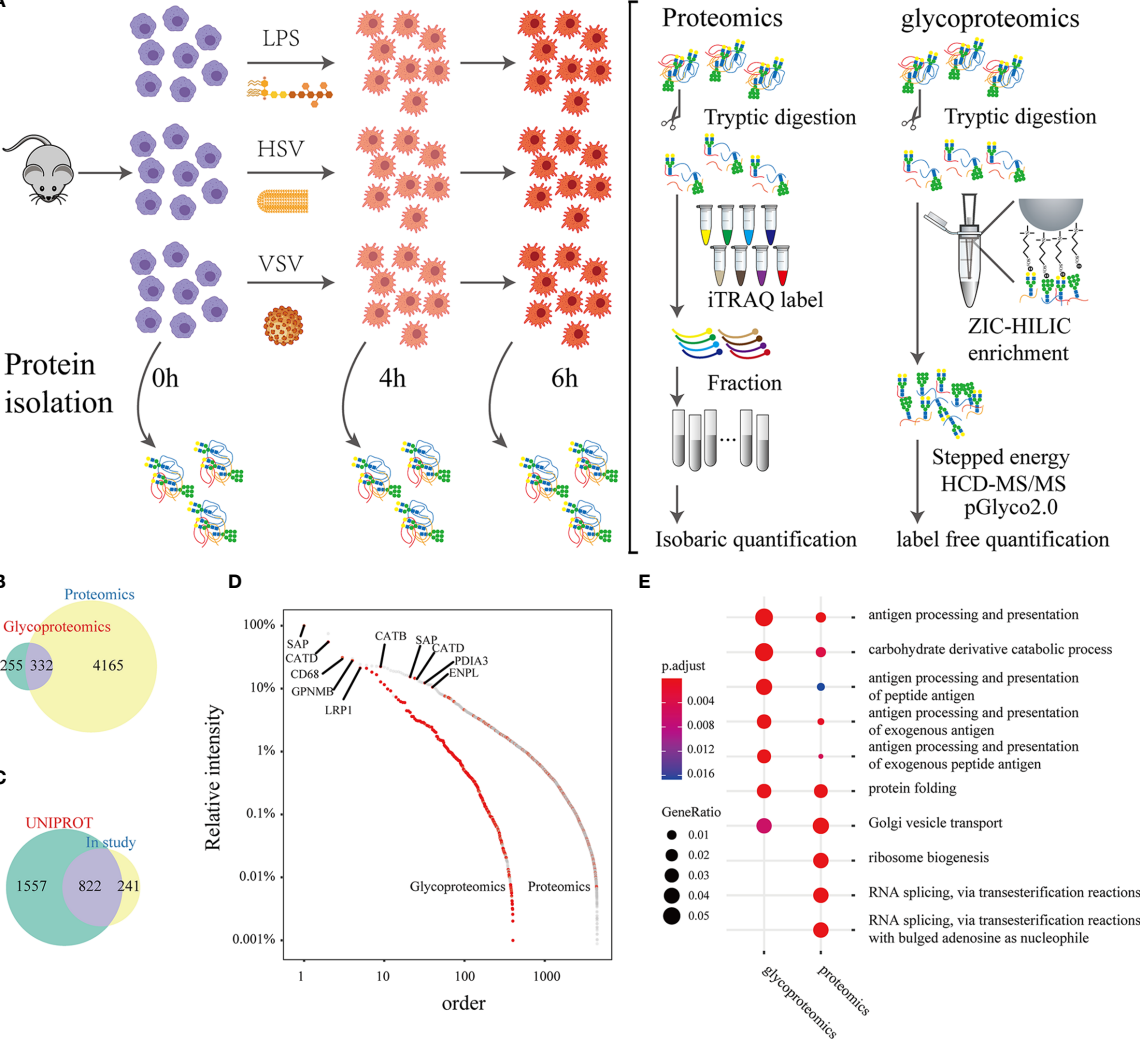
A brief summary of proteomic and glycoproteomic analyses. Experimental workflow: Peritoneal macrophages were extracted from mice and stimulated with LPS, HSV or VSV. Lysates were collected from macrophages at 4 hours and 6 hours after stimulation **(A)**. The Venn plot shows the proteins identified by proteomics and glycoproteomics **(B)**. The Venn plot of glycosylation sites that were identified in this study and recorded in the UNIPROT database **(C)**. Abundance distribution of the proteins and glycoproteins identified by the proteomic and glycoproteomic analyses (the relative intensity of the proteins was calculated by the maximum intensity in either the proteome or glycoproteome) **(D)**. Gene Ontology analysis of the proteomic and glycoproteomic data **(E)**.

We integrated multiple technologies to quantify the N-glycoproteome and proteome and to study the changes in macrophages in response to different stimuli (LPS, HSV, VSV) at different times (0 hours, 4 hours, 6 hours) (**Figure 1A**). In quantitative proteomic analyses, peptides were labeled with iTRAQ 8-plex reagent and analyzed by LC-MS/MS. Intact N-glycopeptides were quantified using a label-free method and were directly identified with pGlyco 2.0. A total of 4497 proteins were identified by proteomic analysis (**Table S1**), and 587 glycoproteins were identified by glycoproteomic analysis. Three hundred thirty-two glycoproteins were identified in both proteomic and glycoproteomic analyses (**Figure 1B**). In three biological replicates, we identified 8326 intact N-glycopeptides and 526 N-glycan components from 1239 peptides on 587 glycoproteins (**Figure S1A** and **Table S2**). The correlation coefficient of biological replicates in the same group was above 0.85 (**Figure S1B**).

Among the N-glycosylation sites identified in our study, 822 sites were recorded in the UNIPROT database, and 241 were uniquely identified in this study (**Figure 1C**). SAP, CATD, and CD68 had relatively higher abundances in both the proteomic and glycoproteomic analyses (**Figure 1D**). Gene Ontology analysis displayed a remarkable enrichment in immunity-related pathways (such as antigen processing and presentation) in the both glycoproteomics and proteomics (**Figure S2**). The gene sets from the proteomic analysis with strong enrichment in RNA splicing and ribosome biogenesis were eliminated in the glycoproteomic analysis (**Figure 1E**). Next, we grouped the GO terms of the identified glycoproteins into a correlation network by calculating common genes. The GO terms were cytokine secretion, antigen presentation, phagocytosis and other immune processes (**Figure S3**). In summary, these results indicated that glycoproteins were focused on immune function.

### Global Analysis of Intact N-Glycopeptides in Murine Macrophages

**Figure 2:**
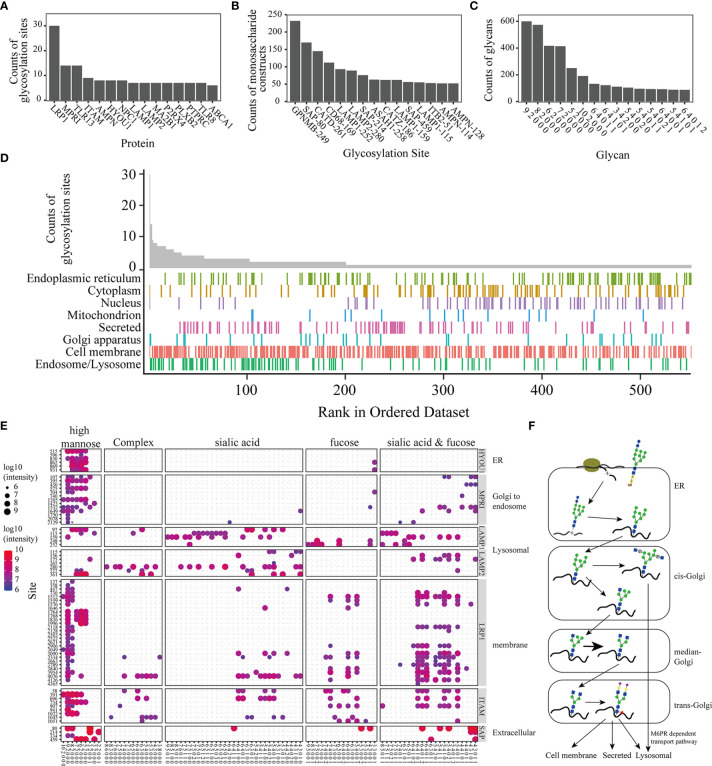
Analysis of the precise N-glycoproteome. Bar plot of the top 15 glycoproteins with most glycosylation sites **(A)**, the glycosylation sites with the top 15 monosaccharide constructs **(B)**, and the monosaccharide construction (the ‘9 2 0 0 0’ in the figures represent glycans constituted by 9 Hex, 2 HexNAc, 0 NeuAc, 0 NeuGc, and 0 fucose. The remaining molecules were similarly described) on most glycopeptides **(C)**. The distribution of glycoproteins in different subcellular locations **(D)**. Glycan distribution of the glycoproteins. The size and color of the dot represent the total intensity of the intact glycopeptides **(E)**. Diagram of the glycosylation progress in different subcellular organelles **(F)**.

Approximately 63.77% of glycoproteins have one glycosylation site, and 3 glycoproteins have over 10 glycosylation sites. MPR1 and TLR13 were found to have 12 glycosylation sites in this study (**Figure 2A**). LRP1, an endocytosis receptor mainly involved in endocytosis and phagocytosis of apoptotic cells (25), was shown to have 30 glycosylation sites. MPRI and TLR13 had 13 glycosylation sites. MPRI (known as cation-independent mannose-6-phosphate receptor) mediates the transport of phosphorylated lysosomal enzymes from the Golgi apparatus and cell surface to lysosomes. TLR13 is a member of the Toll-like receptor family, is mainly found in lysosomes, and recognizes bacterial S23 ribosomes (26). Over 100 different glycans were identified on the glycosylation sites of GPNMB (N249), SAP (N80), CATD (N261), and CD68 (N169) (**Figure 2B**). The distribution of the glycosites and site-specific glycans showed the microheterogeneity of protein N-glycosites. The high mannose N-glycan monosaccharide was the most popular N-glycan attached to the glycopeptides (**Figure 2C**). KEGG enrichment analysis on proteins modified with both sialic acid and fucose demonstrated that lysosome and phagosome related processes are shown significantly enriched in sialylated and fucosylated glycoproteins. (**Figure S4**). The distribution of glycoproteins in different subcellular locations demonstrated that proteins on the cell membrane were widely glycosylated, lysosomal glycoproteins tended to have multiple glycosites, and most of these proteins had more than 2 sites (**Figure 2D**).

Glycosylation enzymes operate predominantly in the endoplasmic reticulum (ER) and Golgi, a highly compartmentalized membrane-bound environment (27). **Figures 2E** and **S5** shows the different subcellular locations of these glycoproteins; in other words, the glycans on diverse proteins tended to be different. Glycan processing from the ER to the Golgi gives rise to three main classes of glycans: high-mannose, hybrid and complex glycans (**Figure 2F**).

### Variated Processes of the N-Glycoproteome in Activated Murine Peritoneal Macrophages

**Figure 3:**
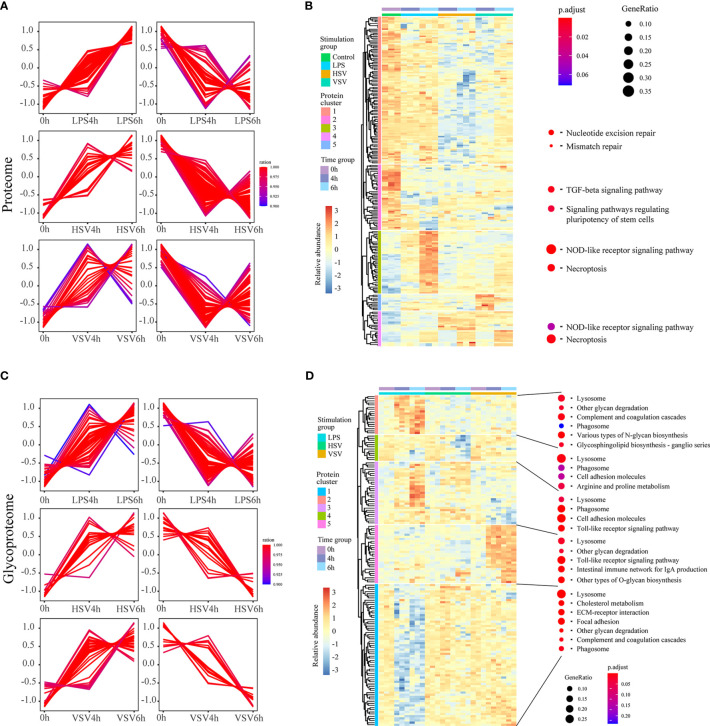
Regulatory groups and functional differences. Proteins with consistently upregulated and downregulated expression in proteomic **(A)** and glycoproteomic profiling **(C)** under these three different stimulations. These continuously changed proteomes were further clustered in a heatmap and are presented with related GO terms **(B, D)**.

Proteins and glycoproteins with consistently upregulated and downregulated expression under stimulation with LPS, HSV and VSV were clustered using fuzzy C-means clustering (**Figures 3A, C**). Then, they were clustered in a heatmap (**Figures 3B, D**).

In the proteome, the expression levels of proteins in the ‘TGF-beta signaling pathway’ and ‘signaling pathways regulating pluripotency of stem cells’ categories were downregulated, and those in ‘NOD-like receptor signaling pathway’ and ‘necroptosis’ were upregulated under stimulation with LPS, HSV or VSV (**Figure 3B**). In the glycoproteome, the levels of glycoproteins in the ‘complement and coagulation cascades’, ‘glycosphingolipid biosynthesis – ganglio series’ and ‘lysosome’ pathways were upregulated under LPS stimulation. The categories ‘Toll-like receptor signaling’ and ‘phagosome’ were enriched under stimulation with LPS, HSV or VSV (**Figure 3D**). In brief, the variation in the proteins in macrophages under LPS stimulation was different from that under stimulation with HSV and VSV in both the proteome and glycoproteome. The proteomic alterations under HSV and VSV stimulation showed a similar pattern (**Figure 3B**), while the glycoproteome changes under HSV stimulation shared a similar pattern with those under LPS stimulation (**Figure 3D**). The N-glycoproteomic analysis also indicated that the Toll-like receptor pathway changed during these three conditions.

**Figure 4:**
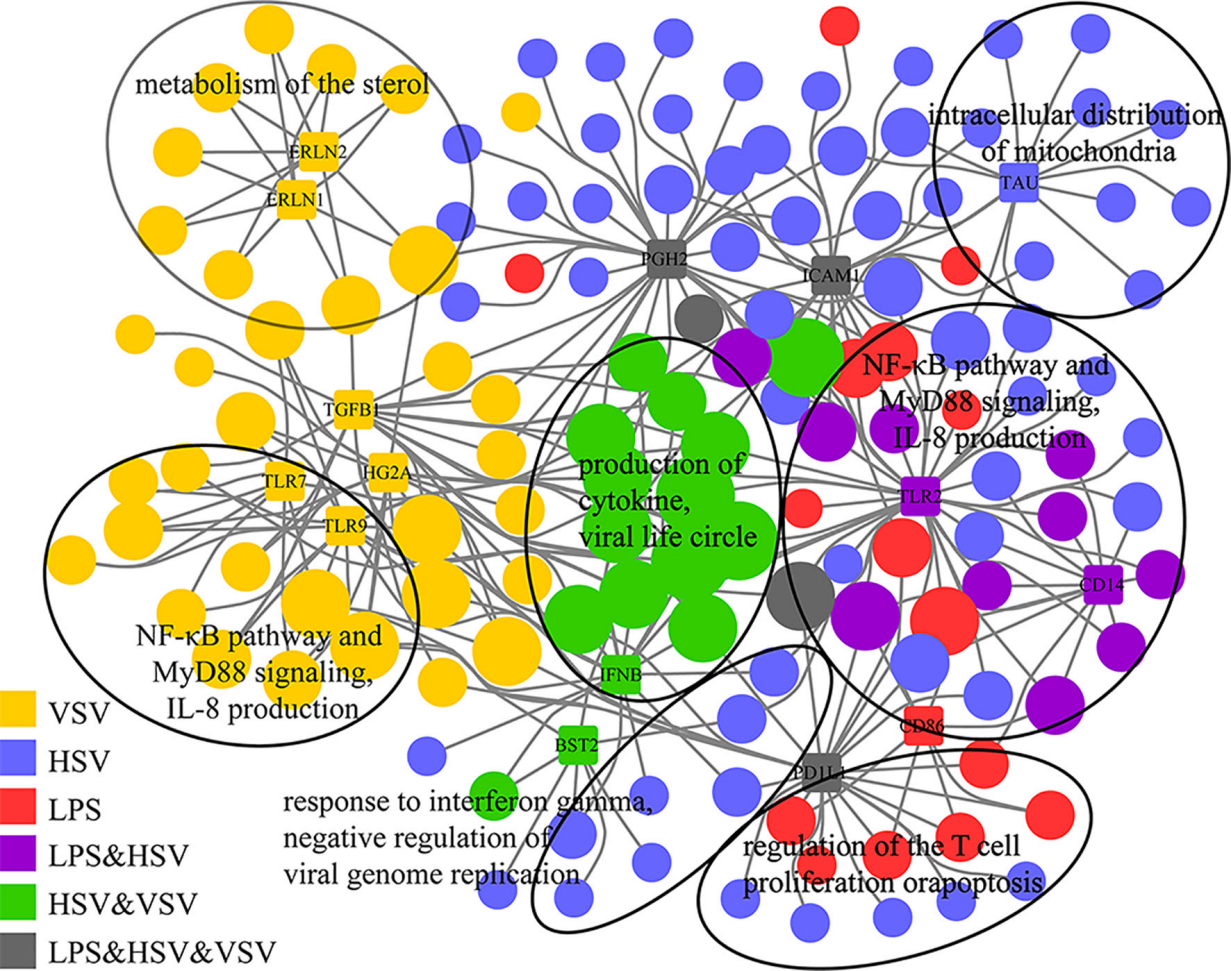
Gene-concept networks of glycoproteomics. Gene-concept network of significant GO terms enriched by consistently upregulated glycoproteins (grouped in **Figure 3C**) and their associations with top-related glycoproteins. The glycoproteins are represented by colored rectangles with the protein names in the center. Enriched GO terms are represented by colored circles. Colors illustrates changed glycoproteins under different stimulations (single or combinational). The size of each circle is proportional to the number of belonging to multiple annotation categories.

The gene-concept network showed the enriched GO terms of glycoproteins with consistently upregulated expression and their relationship with Toll-like receptors (**Figure 4** and **Table S3**). TLR7 and TLR9 were related to VSV stimulation, while TLR2 and CD14 were related to LPS and HSV treatment (**Figure 4**). TLR7, TLR9, TLR2 and CD14 were related to the NF-κB pathway and IL-8 production. IFNB (interferon beta) was induced under HSV and VSV stimulation, which was related to virus infection. The enrichment networks showed that Toll-like receptors (TLR2, CD14, TLR7) were important in these different stimulations. These glycoproteins are pattern recognition proteins that trigger the innate immune response.

### Glycosylation Regulates the Subcellular Localization of Glycoproteins and the Expression of Cytokines

**Figure 5:**
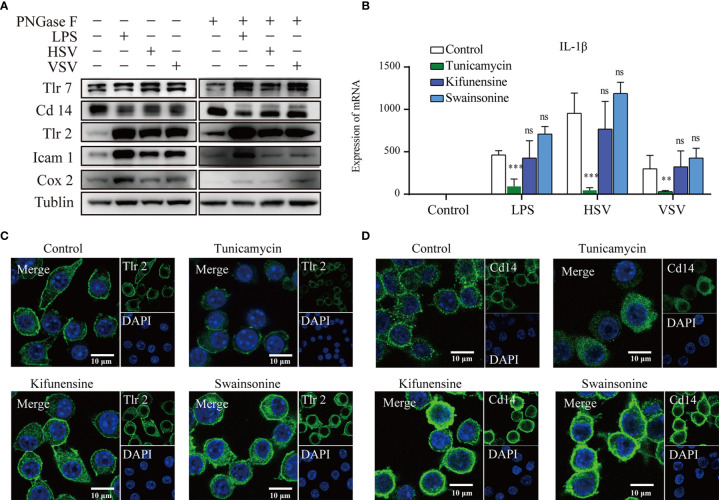
Glycosylation inhibitors altered the membrane location. Western blots of Tlr7, Cd14, Tlr2, Icam1, Cox2, and tubulin with or without PNGase F treatment **(A)**. The mRNA expression of IL-1β **(B)** in RAW264.7 cells under different stimulations after treatment with different glycosylation inhibitors (ns, no significant vs the control group, **p value < 0.01, ***p value < 0.001). Confocal images of Tlr2 **(C)** and Cd14 **(D)** after treatment with different glycosylation inhibitors. Scale bar 10µm.

To confirmed the expression of the glycoproteins, we performed western blot of Cd14, Tlr2, Tlr7, Icam1, and Cox2 (**Figure 5A**). These results showed that Tlr2, Cox2 and Icam1 display upregulated expression under LPS, HSV and VSV stimulation. Tlr7 and Cd14 had no significant change under the stimulation (**Figure 5A**). Therefore, we checked the co-location of the Tlr7 and Lamp1 (lysosomal membrane markers) (**Figures S6A–D**). The results of the confocal image showed that co-location of the Tlr7 with Lamp1 was significant raised under the stimulation of the VSV (**Figure S6E**).

To explore the effect of glycosylation on the subcellular location of glycoproteins, we applied an N-glycosylation inhibitor to the RAW264.7 macrophagic cell line. Tunicamycin is a UDP-GlcNAc analog that can inhibit the transfer of precursor oligosaccharides to the nascent polypeptide chain. Kifunensine and swainsonine are alkaloids that inhibit endoplasmic reticulum mannose I and Golgi alpha mannose II, respectively. The response to ConA lectin in RAW264.7 lysates was lower after treatment with tunicamycin but higher after treatment with kifunensine and swainsonine. PHA-E lectin resulted in a higher response after treatment with tunicamycin and a higher response after treatment with kifunensine and swainsonine (**Figure S7**). The cell membrane localization of Tlr2 was decreased after treatment with tunicamycin, while that in the cytoplasm was also increased after treatment with kifunensine and swainsonine (**Figure 5C**). The cell membrane localization of Cd14 was also decreased in the tunicamycin treatment group, while the fluorescence and cytoplasmic location of Cd14 was increased in the kifunensine and swainsonine treatment groups (**Figure 5D**).

Since the subcellular localization of Toll-like receptors was significantly changed after treatment with different glycosylation inhibitors, we explored the cytokine expression of RAW264.7 cells under stimulation with different pathogens (LPS, HSV, VSV) and different glycosylation inhibitors. Under LPS, HSV and VSV stimulation, the expression of IL-1β was suppressed by tunicamycin treatment (**Figure 5B**). Under VSV stimulation, the expression of IFN-γ was significantly upregulated after tunicamycin treatment. Expression of TNFα demonstrates no significant changes under the treatment of different glycosylation inhibitors (**Figure S8**).

**Figure 6:**
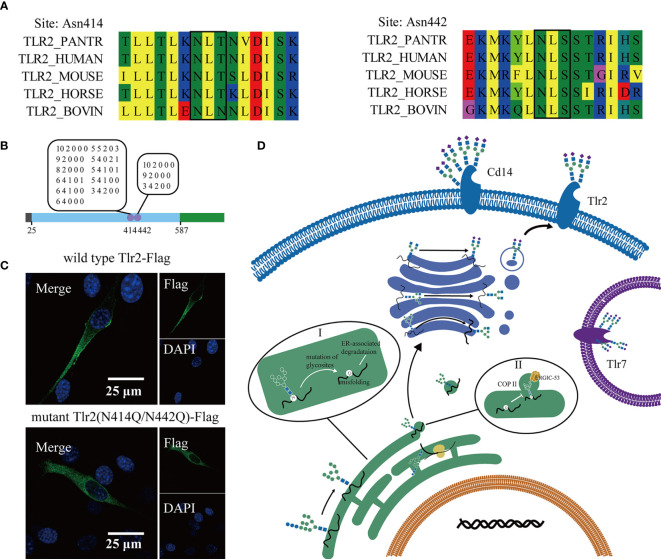
Mutation of the glycosylation sites in Tlr2 altered its membrane location. Multiple sequence alignment of Tlr2 (N-glycosites) with other species **(A)**. Diagram of the Tlr2 sequence and the identified glycosylation sites and glycans **(B)**. Confocal image of 3T3 cells transfected with wild-type Tlr2 and glycosylation mutant Tlr2. Scale bar 25µm **(C)**. A schematic diagram of the subcellular location of the Toll-like pathway and vesicular transport; Aberrant glycosylation lead to misfolding of glycoprotein and ER associated degradation (I); ERGIC-53 bound to oligomannose oligosaccharide promote ER-Golgi apparatus COP II trafficking vesicle formation (II) **(D)**.

Previous analysis indicated that the glycosylation of Toll-like receptors is important in the response to pathogen stimulation. Alignment of the Tlr2 amino acid sequence of different species showed that the glycosylation site identified in this study was conserved in the species listed below (**Figure 6A**). The glycan on the two-glycosylation site was a high-mannose and complex N-glycan with terminal modification of sialic acid (**Figure 6B**). According to a previous study, glycosyltransferase is present in the ER and Golgi apparatus. High mannose N-glycan types are transferred to nascent peptides in the ER, and complex N-glycans progress to the Golgi apparatus. This result indicated that Tlr2 was present not only on the cell membrane but also on some part of the receptor stored in the ER. To confirm the effect of glycosylation on subcellular location, we reconstituted two plasmids expressing wild-type Tlr2-Flag and N-glycosylation site-mutated Tlr2 (N414Q/N442Q)-Flag (Supporting information). The Flag tag from the NIH-3T3 cells transfected with the wild-type Tlr2 displayed predominant membrane localization (**Figure 6C**). The N-glycosylation site mutation significantly reduced membrane localization. This finding indicated that the N-glycan on Tlr2 is important in its transport to the cell membrane.

## Discussion

Exploration of the function of glycosylation in the immune response has indicated the urgent need for high-throughput intact glycopeptide characterization. In this study, large-scale intact glycopeptide characterization was performed using the pGlyco 2.0 workflow. We identified 8236 intact glycopeptides in 587 glycoproteins. GO analysis showed that glycoproteins identified in macrophages are related to many important immune processes. Integrated proteome and glycoproteome analysis demonstrated that the Toll-like receptor pathway was dramatically changed under LPS, HSV and VSV stimulation. SAP, CATD, and CD68 had relatively higher abundances in both the proteomic and glycoproteomic analyses (**Figure 1D**). SAP is also called prosaposin, a kind of lysosomal component that participates in and stimulates the hydrolysis of glucosylceramide (28). CATD (29) and CD68 (30) endoproteases are ubiquitously distributed in lysosomes. Moreover, aberrant glycosylation of Tlr2 and CD14 abolishes its cell membrane location (**Figures 5C, D**).

Global profiling of the precision N-glycoproteomics in macrophages revealed a process with high control and precision during protein transport. N-glycosylation is initiated in the endothelial apparatus, and the nascent peptides are attached to high-mannose glycans. High mannose types are attached to all N-glycopeptides at the beginning of this process. The counts of the peptides that were observed to be modified with high mannose-type N-glycans are shown (**Figure 2C**). The subsequent processing of glycans occurs in the Golgi. The glycan types on the proteins were related to their transport progress and subcellular location. Glycans from different proteins located in different subcellular organelles tend to be modified by different glycosyltransferases and glycohydrolases (31). In this research, the glycans on different glycoproteins (reported in different subcellular locations) tended to be modified with related types of N-glycans (**Figure 2E**). For example, HYOU1 (hypoxia upregulated protein 1), located in the ER and participating in protein folding, was found to have a large number of high mannose-type N-glycans. MPRI (mannose-6-phosphate receptors), which mediates the M6PR-dependent transport pathway (Golgi to endosome) (32), preferred hybrid-type N-glycans. LAMP1 and LAMP2, known as lysosomal membranes, were shown to have complex-type N-glycans with more oligosaccharides (7 5 1 0 0, 6 5 1 0 1 and so on). LRP1 and ITAM, located on the cell membrane, were identified with hybrid-type N-glycans and complex-type N-glycans (Hex = 5 or 6, HexNAc = 3 or 4). SAP is a secreted protein. This protein was shown to be modified with hybrid-type N-glycans and complex-type N-glycans. The glycome profiling of different glycoproteins showed that glycans were regulated by the progress of trafficking. Notably, the distribution of glycoproteins in different subcellular locations indicated that proteins in the lysosomal and cell membrane showed heavy glycosylation (**Figure 2D**). Some proteins on the cell membrane and lysosomes have transmembrane structures (such as LRP1, LAMP1, and LAMP2). Glycosylation sites mostly exist on the outer membrane motif (33). Lysosomal membrane proteins (LMPs) are usually highly glycosylated, probably forming a continuous glycoprotein layer at the luminal side of the lysosomal membrane (34).

To further explore the changes after stimulation with different pathogens (LPS, HSV, VSV), we quantified the intact glycopeptides in a label-free manner. Glycoproteins were significantly changed under pathogenic stimulation (LPS, HSV, VSV). The Toll-like receptor pathway was significantly changed under all three conditions (**Figure 3D**). Toll-like receptors are important PRRs (pattern recognition receptors) located at the cell membrane or lysosome. These molecules mainly participate in PAMP (pathogen-associated molecular pattern) recognition and activation of the immune pathway. The receptors (Tlr2, Tlr7, Cd14, Tlr8, Tlr9, and Tlr13) in the Toll-like receptor pathway are glycoproteins. The activation of innate immune receptors by PAMPs is central to host defense against infection. Significantly, the expression of these receptors (Cd14, Tlr2, Tlr7, and Tlr9) was upregulated under the different stimulations (**Figure 4**). This result indicated that these glycoproteins are regulated by pathogenic stimuli. Previous studies have shown that Cd14 is responsible for LPS recognition (35) and that Tlr2 is responsible for HSV recognition (36, 37). Tlr7 is the lysosomal receptor and recognizes single-stranded RNA (38), and VSV is an ssRNA virus (39). All of the above results indicated that glycosylation of these proteins is important for the macrophage response to pathogens and that glycosylation may affect the stability and localization of these proteins.

According to our data, the biosynthesis of N-glycans on glycoproteins was highly ordered and related to their stability and location. We hypothesize that glycosylation on Toll-like receptors influences their location. Inhibitors of N-glycan (tunicamycin, kifunensine, swainsonine) altered cytokine induction and the membrane location of Tlr2 and Cd14. Tunicamycin reduced the cell surface location of both Cd14 and Tlr2, while kifunensine and swainsonine increased the expression of Cd14 and Tlr2 in the cytoplasm. A reconstitution plasmid with wild-type Tlr2 and mutated Tlr2 (N414Q/N442Q) genes was transfected into the NIH-3T3 cell line. Confocal imaging showed that the N-glycosylation site mutation of Tlr2 reduced its membrane expression (**Figure 6C**). The result indicated that the stability and trafficking of Tlr2 was dependent of N-glycan synthesis in ER, independent of complex type N-glycan processing in Golgi apparatus. Previous studies showed that N-glycosylation is necessary for proper folding of proteins for them to exit the ER, the lack of N-glycans could result in ER retention, which of course would prevent trafficking to any membrane (40). Some intracellular lectins were essential for the formation of COPII-coated transport vesicles, like ERGIC-53 (recognize oligomannose type glycan), bound the glycan on the protein (41) (**Figure 6D**). Subcellular localization determines the environments in which proteins operate. As pattern recognition receptors, Toll-like receptors can bind to conserved pathogen-associated molecular patterns (PAMPs) and trigger the immune response. Different PAMPs will appear at different subcellular locations, and changes in these receptors will increase the distance between receptors and PAMPs. Thus, we provided a valuable study of glycosylation on glycoproteins in macrophages.

## Data Availability Statement

The mass spectrometry N-glycoproteomics data have been deposited to the ProteomeXchange Consortium via the PRIDE partner repository with the dataset identifier PXD026629.

## Ethics Statement

The animal study was reviewed and approved by Department of laboratory animal science, Fudan University.

## Author Contributions

LY conducted experiments and data analysis and drafted the manuscript. TG revised the manuscript and analyzed the data. HS conducted experiments and revised the manuscript. JP performed the confocal image acquisition. LZ performed the label free quantification. QZ revised the manuscript. YH revised the manuscript. ZH performed experiments. ZP performed experiments. PY contributed to project conception and design. LL conducted experiments and drafted the manuscript. HY contributed to project conception and design. All authors contributed to the article and approved the submitted version.

## Funding

This work was supported by National Key Research and Development Program (Grant No. 2018YFA0507501) and National Natural Science Foundation of China (Grant No. 81872258, 82073077,81827901).

## Conflict of Interest

The authors declare that the research was conducted in the absence of any commercial or financial relationships that could be construed as a potential conflict of interest.

## Publisher’s Note

All claims expressed in this article are solely those of the authors and do not necessarily represent those of their affiliated organizations, or those of the publisher, the editors and the reviewers. Any product that may be evaluated in this article, or claim that may be made by its manufacturer, is not guaranteed or endorsed by the publisher.

## In Memoriam

This paper is dedicated to the memory of Professor YANG Pengyuan (deceased on May 31, 2021). Professor Yang was an accomplished scientist, who dedicated his career to the study of mass spectrum and proteomics. He was not only an inspiring mentor to his students but also a beloved leader of the whole laboratory staff.
