# Supplementary material for: Precision N-Glycoproteomic Profiling of Murine Peritoneal Macrophages After Different Stimulations
Source: Front Immunol. 2021 Aug 17;12:722293. doi: 10.3389/fimmu.2021.722293 (PMC8416091; doi:10.3389/fimmu.2021.722293)
Supplement: Supplementary file 4 [file DataSheet_4.docx]

**Precision N-Glycoproteomic Profiling of Murine Peritoneal Macrophages After Different Stimulations**

Lujie Yang, Tianqi Gong, Huali Shen, Jiangnan Pei, Lei Zhang, Quanqing Zhang, Yuanyu Huang, Zuojian Hu, Ziyue Pan, Pengyuan Yang, Ling Lin, and Hongxiu Yu,

1. Institute of biomedicine science & department of chemistry, Fudan University
2. Xiamen University
3. University of California
4. Guanxi medical University

Content

1. Supporting materials and methods
2. Supporting figures
3. Supporting Tables
4. **Supporting materials and methods**

**RNA extraction and RT-PCR**

Lysis the cell with 1 ml Triton. Add 500 ul chloroform, put it upside down and sit on ice for 10 minutes. Centrifuge at 10000 rpm for 10 minutes, and then transfer the supernatant to a new EP tube. Add 500 uL methanol to the supernatant and upside down several times. Then centrifuge at 10000 rpm for 10 minutes. Remove the supernatant and remain the precipitate. Wash the RNA with 75% ethanol 2 times. Dry the alcohol and re-dissolve the RNA with deionized water. Quantitative the concentration of the RNA with Nanodrop 2000. Reverse transcription with Takara’s reverse transcription kit. And qPCR was operated with a qPCR kit according to its user manual.

**LC-MS Analysis of Site-specific N-glycopeptides.**

Enriched glycopeptides were directly analyzed by nano spray LC-MS/MS on an Orbitrap Fusion Tribrid (Thermo Scientific) coupled to an EASY-nano-LC system (Thermo Scientific) without the trap column. For one LC-MS run, 100 μg of crude proteins from mouse tissues were used as starting material (before HILIC enrichment). Samples were loaded onto a C18 column (50 cm x 75 μm i.d.) and were separated at a flow rate of 300 nL/min. Solvent A was 0.1% formic acid in water. Solvent B was acetonitrile with 0.1% formic acid. The gradient was 4 hours in total for complex samples: 2% to 40% in 345 min, an increase to 90% B in 3 min, hold for another 7 min and hold for 2% B for the last 5 min. The parameters for intact glycopeptide analysis were: (1) MS: scan range (m/z) =800-2,000; resolution =120,000; AGC target =200,000; maximum injection time =100 ms; included charge state =2-6; dynamic exclusion after n times, n =1; dynamic exclusion duration = 15 s; and each selected precursor subject to one HCD-MS/MS; (2) HCD-MS/MS: isolation window =2; detector type =Orbitrap; resolution =15,000; AGC target =500,000; maximum injection time =250 ms; collision energy =30%; and stepped collision mode on with an energy difference of ±10% (10% as absolute value in the Orbitrap Fusion).

**Liquid Chromatography-Mass Spectrometry (LC-MS) for iTRAQ-labeled Samples.**

Each fraction from fractionated samples was analyzed on a Triple-TOFTM 4600 system (AB SCIEX, USA). The Triple-TOF 4600 system was equipped with a nano-HPLC (Eksigent Technologies) with a reversed-phase analytical column (Eksigent, C18, 150 mm×75 µm, 3 µm). An electrospray voltage of 2.5 kV versus the inlet of the mass spectrometer was used. The mass spectrometer was operated in information-dependent data acquisition mode to allow automatic switching between MS and MS/MS acquisition. MS spectra were acquired across the mass range of 350–1,250 m/z with an accumulation time of 250 ms per spectrum. Tandem mass spectra were scanned from 100-1,250 m/z in high-sensitivity mode with rolling collision energy. The 25 most-intense precursors in each cycle were selected for fragmentation, with a dynamic exclusion time of 25 s. The iTRAQ-labeled de-glycopeptides were analyzed on an Orbitrap Fusion Tribrid system (Thermo Fisher Scientific, USA). The Orbitrap Fusion was equipped with a Proxeon EASY-nLC II liquid chromatography pump (Thermo Fisher Scientific) involving a reversed-phase analytical column (Thermo Scientific, C18, 150 mm×75 µm, 3 µm). Mobile phase buffer A was composed of water and 0.2% (v/v) formic acid, while mobile phase B was composed of acetonitrile and 0.2% (v/v) formic acid. Samples were loaded onto the column for 3 min at 2 μL/min. The flow rate was 200 nL/min with the following linear gradient: from 2% to 35% B in 155 min, from 35% to 45% B in 13 min, from 45% to 90% B in 3 min, a 1.5-min wash at 90% B, from 90% to 2% B in 0.5 min, and a 7-min re-equilibration at 2% B. Survey scans of peptide precursors from 350 to 1,500 m/z were performed at 120,000 resolution with a 3 × 105 ion count target. Tandem MS was performed as follows: isolation at 2 Th with the quadrupole, HCD fragmentation with a normalized collision energy of 37%, and detection in orbitrap. The MS2 ion count target was set to 2e5 and the max injection time was 150 ms. Precursors with charge states of 2–4 were sampled for MS2. The dynamic exclusion duration was set to 30 s with a 10 ppm tolerance around the selected precursor and its isotopes. Monoisotopic precursor selection was turned on. The instrument was run in top speed mode with 3 s cycles.

**Database Searching for intact N-glycopeptides and iTRAQ-labeled peptides.**

Site-specific glycopeptides were interpreted using the software pGlyco 2.0. Raw MS/MS data were converted to “mgf” format by the revised version of pParse. Parameters for database searching of intact glycopeptide were as follows: mass tolerance for precursors and fragment ions was set as ±5 ppm and ±20 ppm, respectively. The protein databases were from Swiss-Prot (2015_03, mouse, 16,711 entries). Maximal missed cleavage was 3. Fixed modification was carbamidomethylation (C). Variable modifications contained oxidation on Met (M), acetylation on protein Nterm. The N-glycosylation sequon (N-X-S/T, X ≠ P) was modified by changing “N” to “J” (the two shared the same mass). The glycan database was extracted from GlycomeDB (www.glycome-db.org), and the total entries of N-glycan were 7,884 considering NeuGc. Trypsin and protein databases with species of Mus musculus (16,711 entries) were used. Quantification of site-specific glycopeptides were based on the peak areas as previously described.

iTRAQ-labeled peptides were identified with PEAKS search engine against the SwissProt mouse database. A maximum of two missed tryptic cleavages were allowed. For the global proteomic analysis, static modification of carbamidomethyl (C), iTRAQ 8-plex (K), and iTRAQ 8 plex (Nterm) were set, along with dynamic modifications of oxidation (M), and iTRAQ 8-plex (Y). All data were searched as a single batch with peptide FDR set to 1% using Percolator.

**Gene sequences**

The sequence below which labeled red represent the sequence of Tlr2 or Tlr2(N414/442Q), and highlighted with yellow represented nonsense mutation.

Sequences of Tlr2(N414/442Q):

GCTTACTGGCTTATCGAAATTAATACGACTCACTATAGGGAGACCCAAGCTGGCTAGCGTTTAAACGGGCCCTCTAGACTCGAGCGCCACCATGCTACGAGCTCTTTGGCTCTTCTGGATCTTGGTGGCCATAACAGTCCTCTTCAGCAAACGCTGTTCTGCTCAGGAGTCTCTGTCATGTGATGCTTCTGGGGTGTGTGATGGCCGCTCCAGGTCTTTCACCTCTATTCCCTCCGGACTCACAGCAGCCATGAAAAGCCTTGACCTGTCTTTCAACAAGATCACCTACATTGGCCATGGTGACCTCCGAGCGTGTGCGAACCTCCAGGTTCTGATGTTGAAGTCCAGCAGAATCAATACAATAGAGGGAGACGCCTTTTATTCTCTGGGCAGTCTTGAACATTTGGATTTGTCTGATAATCACCTATCTAGTTTATCTTCCTCCTGGTTCGGGCCCCTTTCCTCTTTGAAATACTTAAACTTAATGGGAAATCCTTACCAGACACTGGGGGTAACATCGCTTTTTCCCAATCTCACAAATTTACAAACCCTCAGGATAGGAAATGTAGAGACTTTCAGTGAGATAAGGAGAATAGATTTTGCTGGGCTGACTTCTCTCAATGAACTTGAAATTAAGGCATTAAGTCTCCGGAATTATCAGTCCCAAAGTCTAAAGTCGATCCGCGACATCCATCACCTGACTCTTCACTTAAGCGAGTCTGCTTTCCTGCTGGAGATTTTTGCAGATATTCTGAGTTCTGTGAGATATTTAGAACTAAGAGATACTAACTTGGCCAGGTTCCAGTTTTCACCACTGCCCGTAGATGAAGTCAGCTCACCGATGAAGAAGCTGGCATTCCGAGGCTCGGTTCTCACTGATGAAAGCTTTAACGAGCTCCTGAAGCTGTTGCGTTACATCTTGGAACTGTCGGAGGTAGAGTTCGACGACTGTACCCTCAATGGGCTCGGCGATTTCAACCCCTCGGAGTCAGACGTAGTGAGCGAGCTGGGTAAAGTAGAAACAGTCACTATCCGGAGGTTGCATATCCCCCAGTTCTATTTGTTTTATGACCTGAGTACTGTCTATTCCCTCCTGGAGAAGGTGAAGCGAATCACAGTAGAGAACAGCAAGGTCTTCCTGGTTCCCTGCTCGTTCTCCCAGCATTTAAAATCATTAGAATTCTTAGACCTCAGCGAAAATCTGATGGTTGAAGAATATTTGAAGAACTCAGCCTGTAAGGGAGCCTGGCCTTCTCTACAAACCTTAGTTTTGAGCCAGAATCATTTGAGATCAATGCAAAAAACAGGAGAGATTTTGCTGACTCTGAAACAACTGACCTCTCTTGACATCAGCAGGAACACTTTTCATCCGATGCCCGACAGCTGTCAGTGGCCAGAAAAGATGCGCTTCCTGCAATTGTCCAGTACAGGGATCCGGGTGGTAAAAACGTGCATTCCTCAGACGCTGGAGGTGTTGGATGTTAGTAACAACAATCTTGACTCATTTTCTTTGTTCTTGCCTCGGCTGCAAGAGCTCTATATTTCCAGAAATAAGCTGAAAACACTCCCAGATGCTTCGTTGTTCCCTGTGTTGCTGGTCATGAAAATCAGAGAGAATGCAGTAAGTACTTTCTCTAAAGACCAACTTGGTTCTTTTCCCAAACTGGAGACTCTGGAAGCAGGCGACAACCACTTTGTTTGCTCCTGCGAACTCCTATCCTTTACTATGGAGACGCCAGCTCTGGCTCAAATCCTGGTTGACTGGCCAGACAGCTACCTGTGTGACTCTCCGCCTCGCCTGCACGGCCACAGGCTTCAGGATGCCCGGCCCTCCGTCTTGGAATGTCACCAGGCTGCACTGGTGTCTGGAGTCTGCTGTGCCCTTCTCCTGTTGATCTTGCTCGTAGGTGCCCTGTGCCACCATTTCCACGGGCTGTGGTACCTGAGAATGATGTGGGCGTGGCTCCAGGCCAAGAGGAAGCCCAAGAAAGCTCCCTGCAGGGACGTTTGCTATGATGCCTTTGTTTCCTACAGTGAGCAGGATTCCCATTGGGTGGAGAACCTCATGGTCCAGCAGCTGGAGAACTCTGACCCGCCCTTTAAGCTGTGTCTCCACAAGCGGGACTTCGTTCCGGGCAAATGGATCATTGACAACATCATCGATTCCATCGAAAAGAGCCACAAAACTGTGTTCGTGCTTTCTGAGAACTTCGTACGGAGCGAGTGGTGCAAGTACGAACTGGACTTCTCCCACTTCAGGCTCTTTGACGAGAACAACGACGCGGCCATCCTTGTTTTGCTGGAGCCCATTGAGAGGAAAGCCATTCCCCAGCGCTTCTGCAAACTGCGCAAGATAATGAACACCAAGACCTACCTGGAGTGGCCCTTGGATGAAGGCCAGCAGGAAGTGTTTTGGGTAAATCTGAGAACTGCAATAAAGTCCTCGGTACCAAGCTTAAGTGACTACAAGGATGACGATGACAAGGATTACAAAGACGACGATGATAAGGACTATAAGGATGATGACGACAAATCTAGATAGTTAAACCGCTGATCAGCCTCGACTGTGCCTTCTAGTTGCCAGCCATCTGTTGTTTGCCCCTCCCCCGTGCCTTCCTTGACCCTGGAAGGTGCCACTCCCACTGTCCTTTCCTAATAAAATGAGGAAATTGCATCGCATTGTCT

Sequences of Tlr2:

CCCAAGCTGGCTAGCGTTTAAACGGGCCCTCTAGACTCGAGCGCCACCATGCTACGAGCTCTTTGGCTCTTCTGGATCTTGGTGGCCATAACAGTCCTCTTCAGCAAACGCTGTTCTGCTCAGGAGTCTCTGTCATGTGATGCTTCTGGGGTGTGTGATGGCCGCTCCAGGTCTTTCACCTCTATTCCCTCCGGACTCACAGCAGCCATGAAAAGCCTTGACCTGTCTTTCAACAAGATCACCTACATTGGCCATGGTGACCTCCGAGCGTGTGCGAACCTCCAGGTTCTGATGTTGAAGTCCAGCAGAATCAATACAATAGAGGGAGACGCCTTTTATTCTCTGGGCAGTCTTGAACATTTGGATTTGTCTGATAATCACCTATCTAGTTTATCTTCCTCCTGGTTCGGGCCCCTTTCCTCTTTGAAATACTTAAACTTAATGGGAAATCCTTACCAGACACTGGGGGTAACATCGCTTTTTCCCAATCTCACAAATTTACAAACCCTCAGGATAGGAAATGTAGAGACTTTCAGTGAGATAAGGAGAATAGATTTTGCTGGGCTGACTTCTCTCAATGAACTTGAAATTAAGGCATTAAGTCTCCGGAATTATCAGTCCCAAAGTCTAAAGTCGATCCGCGACATCCATCACCTGACTCTTCACTTAAGCGAGTCTGCTTTCCTGCTGGAGATTTTTGCAGATATTCTGAGTTCTGTGAGATATTTAGAACTAAGAGATACTAACTTGGCCAGGTTCCAGTTTTCACCACTGCCCGTAGATGAAGTCAGCTCACCGATGAAGAAGCTGGCATTCCGAGGCTCGGTTCTCACTGATGAAAGCTTTAACGAGCTCCTGAAGCTGTTGCGTTACATCTTGGAACTGTCGGAGGTAGAGTTCGACGACTGTACCCTCAATGGGCTCGGCGATTTCAACCCCTCGGAGTCAGACGTAGTGAGCGAGCTGGGTAAAGTAGAAACAGTCACTATCCGGAGGTTGCATATCCCCCAGTTCTATTTGTTTTATGACCTGAGTACTGTCTATTCCCTCCTGGAGAAGGTGAAGCGAATCACAGTAGAGAACAGCAAGGTCTTCCTGGTTCCCTGCTCGTTCTCCCAGCATTTAAAATCATTAGAATTCTTAGACCTCAGCGAAAATCTGATGGTTGAAGAATATTTGAAGAACTCAGCCTGTAAGGGAGCCTGGCCTTCTCTACAAACCTTAGTTTTGAGCCAGAATCATTTGAGATCAATGCAAAAAACAGGAGAGATTTTGCTGACTCTGAAAAACCTGACCTCCCTTGACATCAGCAGGAACACTTTTCATCCGATGCCCGACAGCTGTCAGTGGCCAGAAAAGATGCGCTTCCTGAATTTGTCCAGTACAGGGATCCGGGTGGTAAAAACGTGCATTCCTCAGACGCTGGAGGTGTTGGATGTTAGTAACAACAATCTTGACTCATTTTCTTTGTTCTTGCCTCGGCTGCAAGAGCTCTATATTTCCAGAAATAAGCTGAAAACACTCCCAGATGCTTCGTTGTTCCCTGTGTTGCTGGTCATGAAAATCAGAGAGAATGCAGTAAGTACTTTCTCTAAAGACCAACTTGGTTCTTTTCCCAAACTGGAGACTCTGGAAGCAGGCGACAACCACTTTGTTTGCTCCTGCGAACTCCTATCCTTTACTATGGAGACGCCAGCTCTGGCTCAAATCCTGGTTGACTGGCCAGACAGCTACCTGTGTGACTCTCCGCCTCGCCTGCACGGCCACAGGCTTCAGGATGCCCGGCCCTCCGTCTTGGAATGTCACCAGGCTGCACTGGTGTCTGGAGTCTGCTGTGCCCTTCTCCTGTTGATCTTGCTCGTAGGTGCCCTGTGCCACCATTTCCACGGGCTGTGGTACCTGAGAATGATGTGGGCGTGGCTCCAGGCCAAGAGGAAGCCCAAGAAAGCTCCCTGCAGGGACGTTTGCTATGATGCCTTTGTTTCCTACAGTGAGCAGGATTCCCATTGGGTGGAGAACCTCATGGTCCAGCAGCTGGAGAACTCTGACCCGCCCTTTAAGCTGTGTCTCCACAAGCGGGACTTCGTTCCGGGCAAATGGATCATTGACAACATCATCGATTCCATCGAAAAGAGCCACAAAACTGTGTTCGTGCTTTCTGAGAACTTCGTACGGAGCGAGTGGTGCAAGTACGAACTGGACTTCTCCCACTTCAGGCTCTTTGACGAGAACAACGACGCGGCCATCCTTGTTTTGCTGGAGCCCATTGAGAGGAAAGCCATTCCCCAGCGCTTCTGCAAACTGCGCAAGATAATGAACACCAAGACCTACCTGGAGTGGCCCTTGGATGAAGGCCAGCAGGAAGTGTTTTGGGTAAATCTGAGAACTGCAATAAAGTCCTCGGTACCAAGCTTAAGTGACTACAAGGATGACGATGACAAGGATTACAAAGACGACGATGATAAGGACTATAAGGATGATGACGACAAATCTAGATAGTTAAACCGCTGATCAGCCTCGACTGTGCCTTCTAGTTGCCAGCCATCTG

1. **Supporting figures**


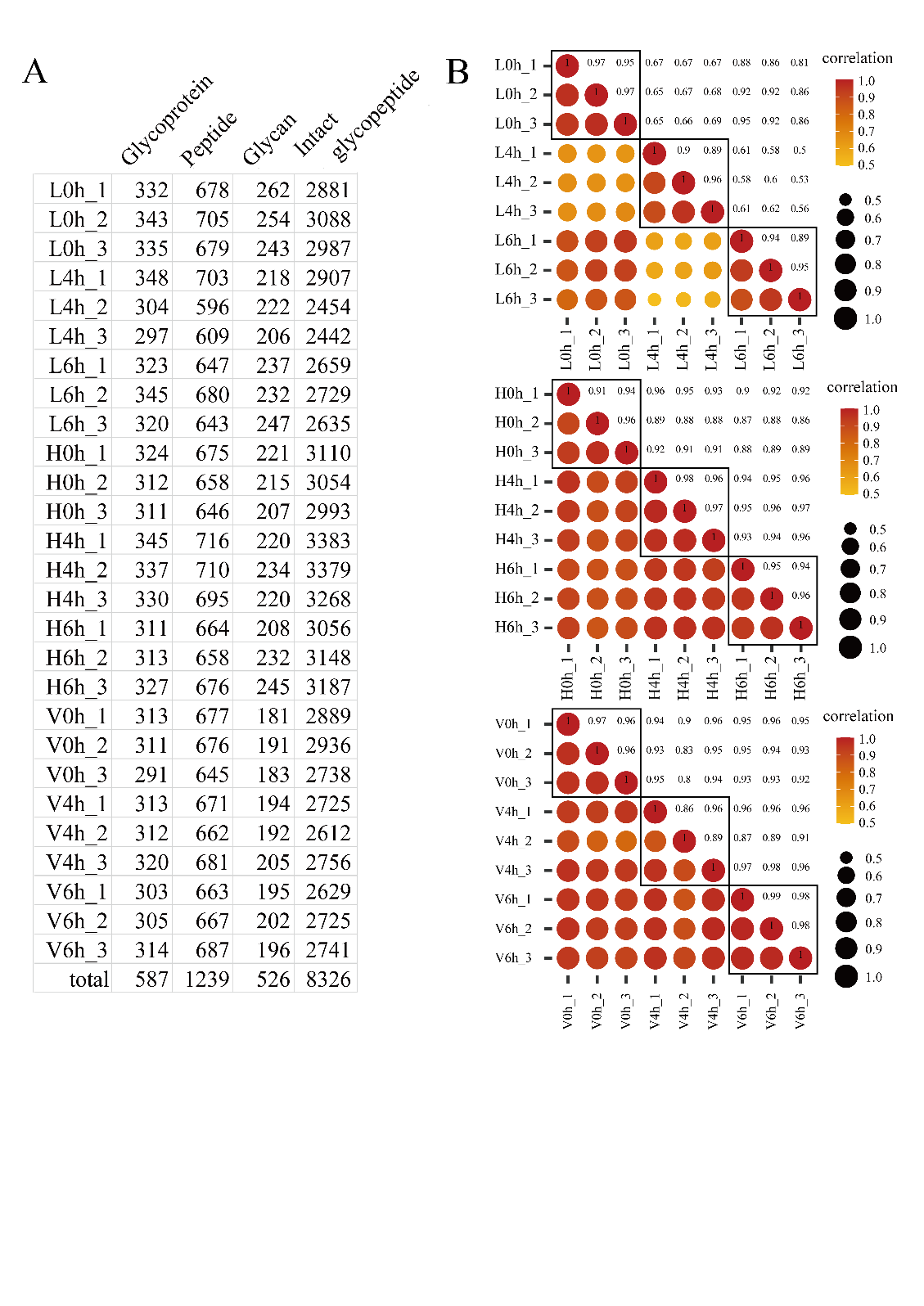


Figure S1. (A) The count of unique glycoproteins, glycopeptides, monosaccharide construction and intact glycopeptides. (B) The correlation between the each run, the color and size were related to the correlation.


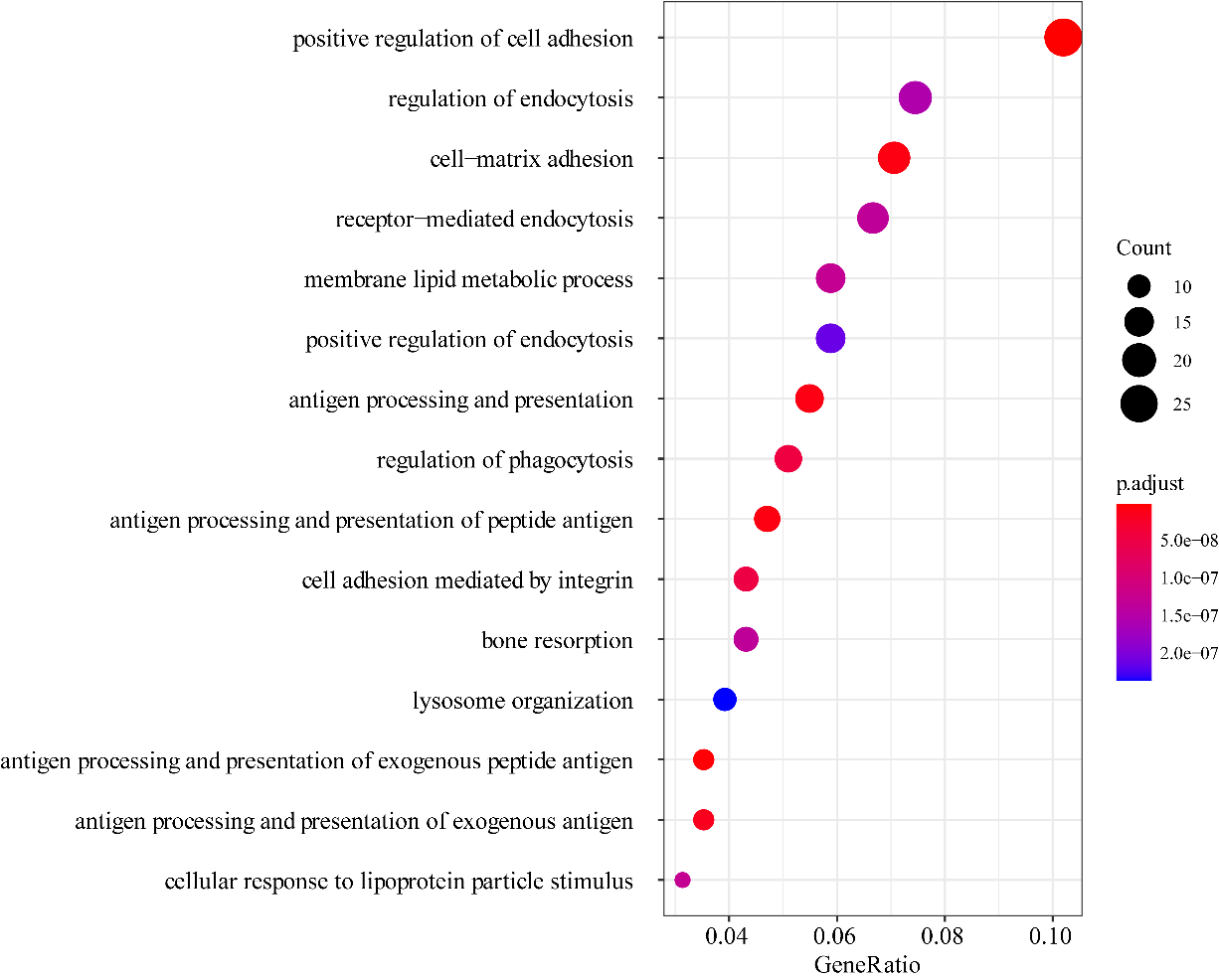


Figure S2 GO terms enriched of overlapped molecules identified in proteome and glycoproteome dataset.


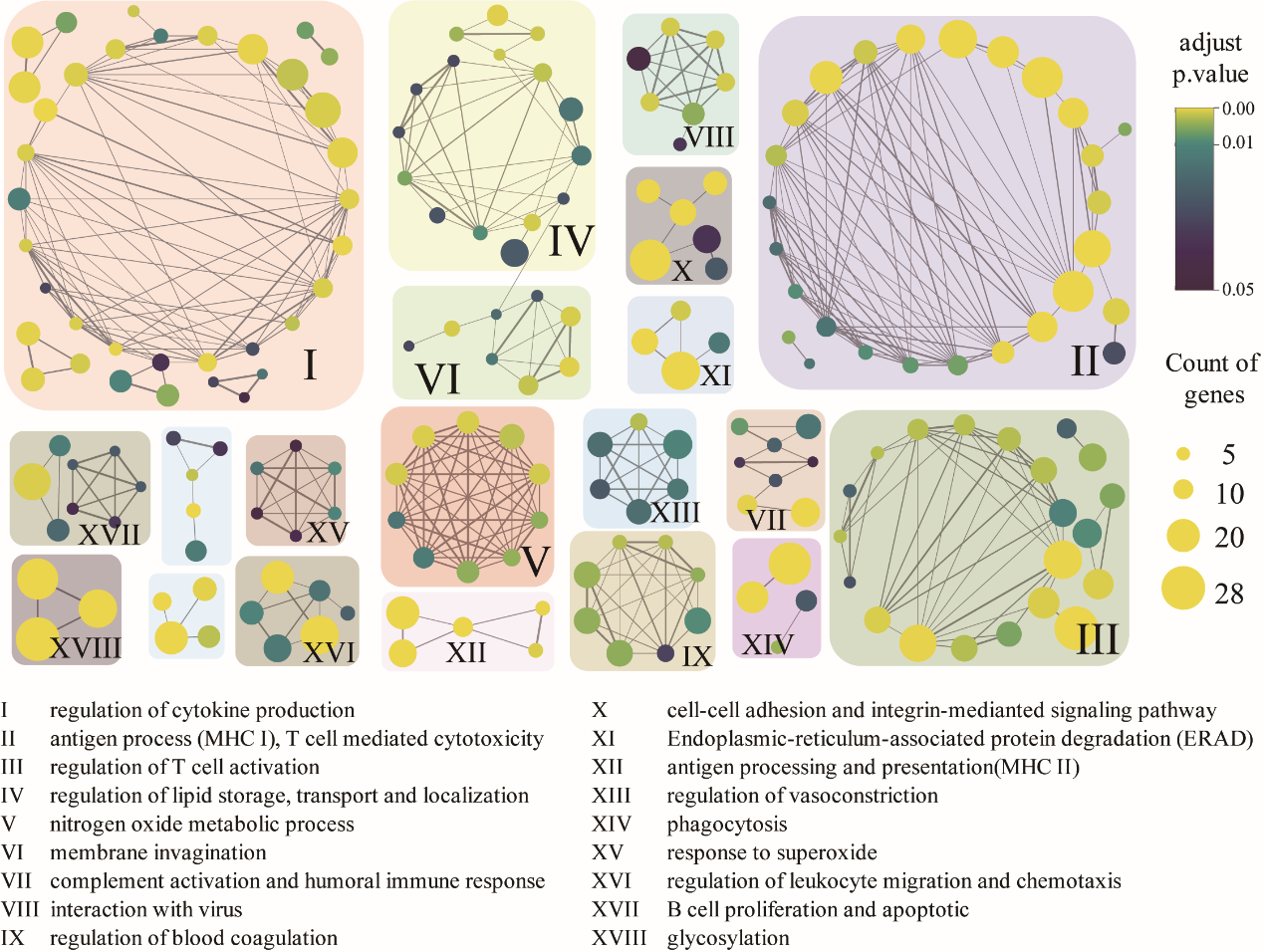


Figure S3 Correlation network of GO terms of the glycoprotein identified in murine peritoneal macrophage. The color of the point represents the -log 10 adjusted p value, the redder the color, the more significant the term. The size of the point represents the counts of glycoproteins enriched in the term. The description of each GO group is listed at the bottom of the figure.


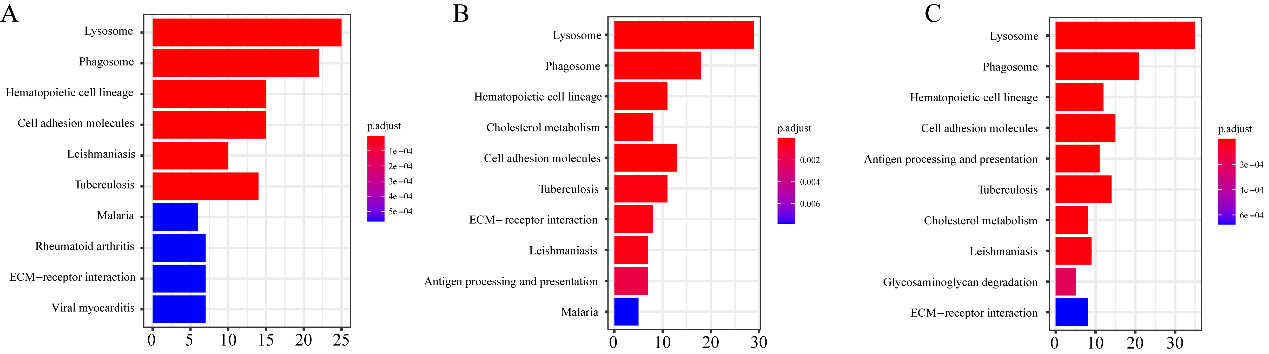


Figure S4 KEGG enrichment analysis on proteins modified with both sialic acid and fucose (A), with only sialic acid not fucose (B), and with only fucose not sialic acid (C).


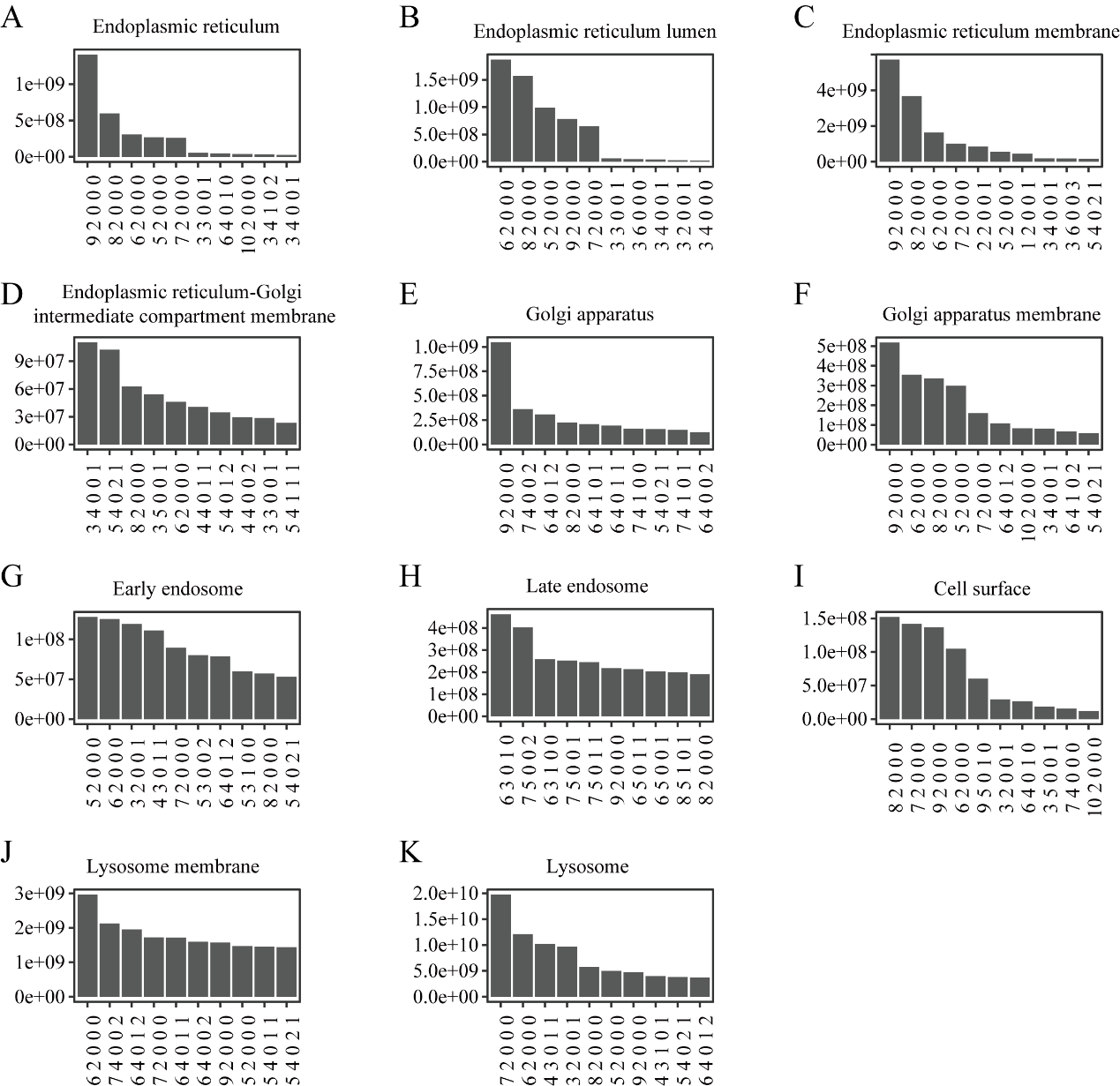


Figure S5. Intensity barplot of N-glycans on the proteins which located in endoplasmic reticulum (A), endoplasmic reticulum lumen (B), endoplasmic reticulum membrane (C), endoplasmic reticulum-Golgi intermediate compartment membrane (D), Golgi apparatus (E), Golgi apparatus membrane (F), early endosome (G), late endosome (H), cell surface (I), lysosome membrane (J) and Lysoosme (K).


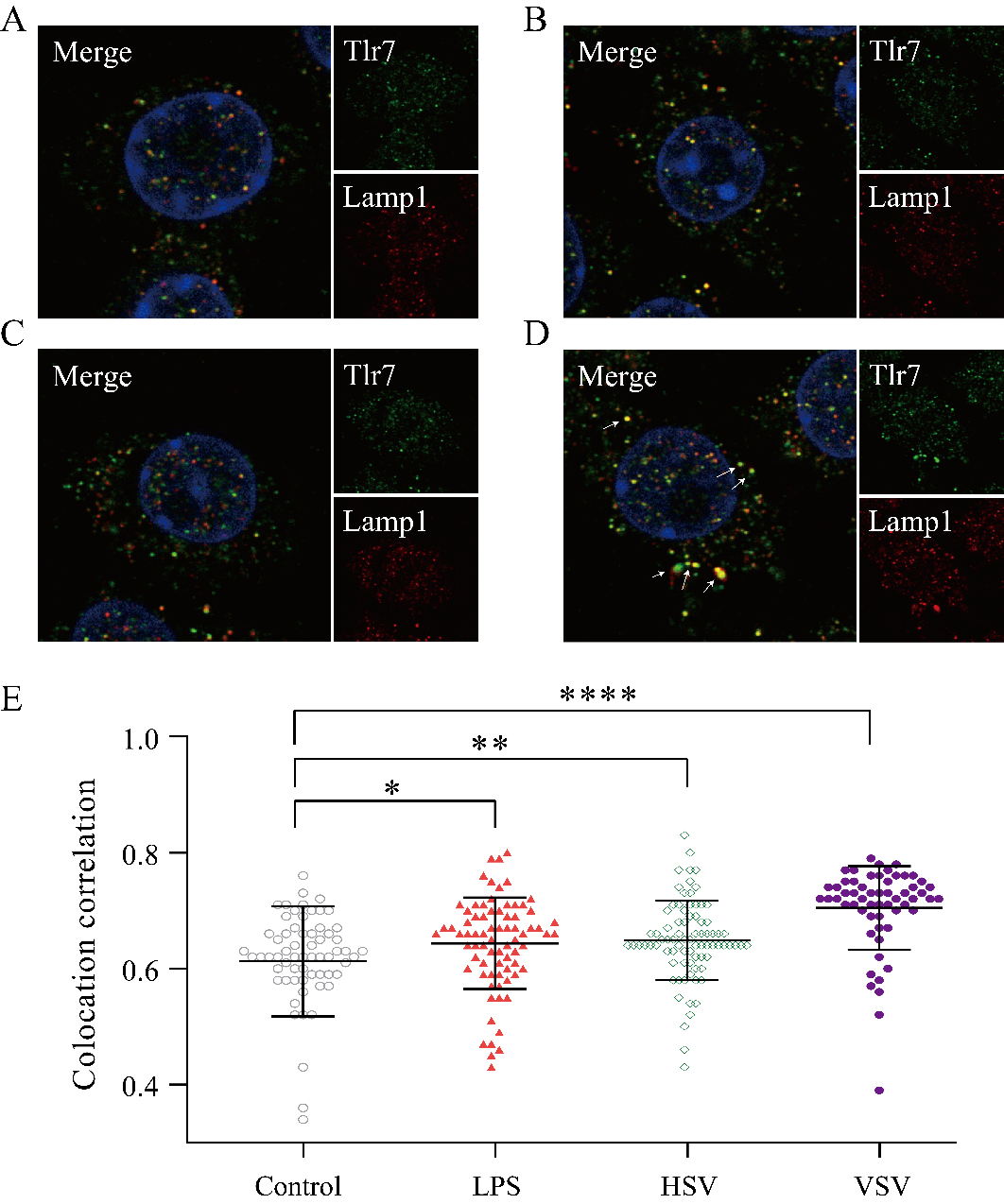


Figure. S6 Representative confocal images Tlr7 and Lamp1 in RAW264.7 (A), after the stimulation of LPS (B), HSV (C) and VSV (D). The correlation coefficient of the Tlr7 and Lamp1 of each cell was calculated by Fuji software and presented in dot plot (***, p<0.001) (E).


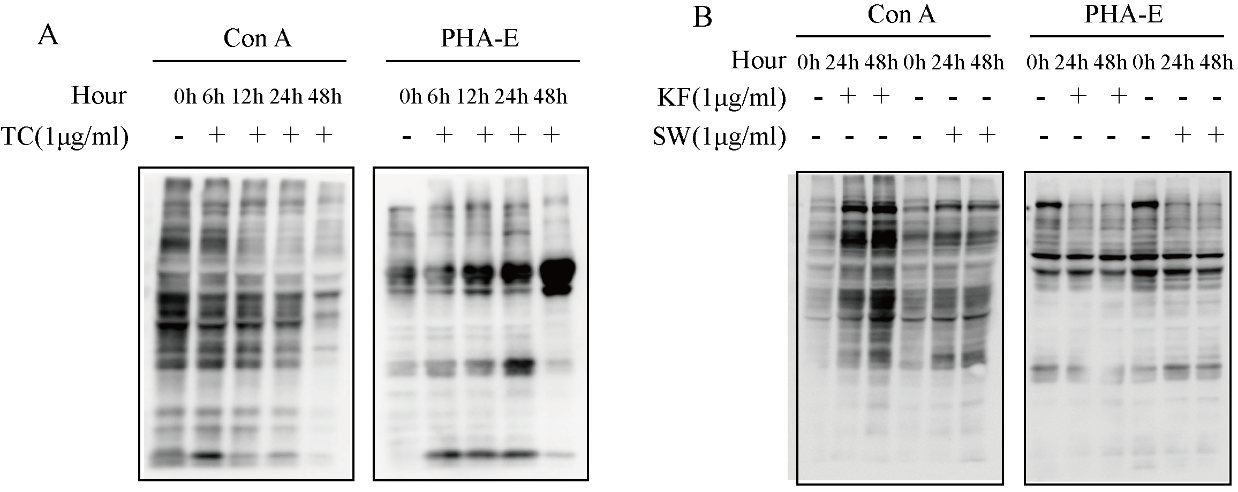


Figure S7. RAW264.7 were treated with tunicamycin (TC) at a concentration of 1 μg/ml in DMEM, the cell lysate was collected at 6, 12, 24 and 48 hours. The lectin blots performed with Con A and PHA-E (A). RAW264.7 were treated with kifunensine and swainsonine at a concentration of 1 μg/ml in DMEM. The lectin blots of lysate collected at 24 and 48 hours was performed with ConA and PHA-E (B).


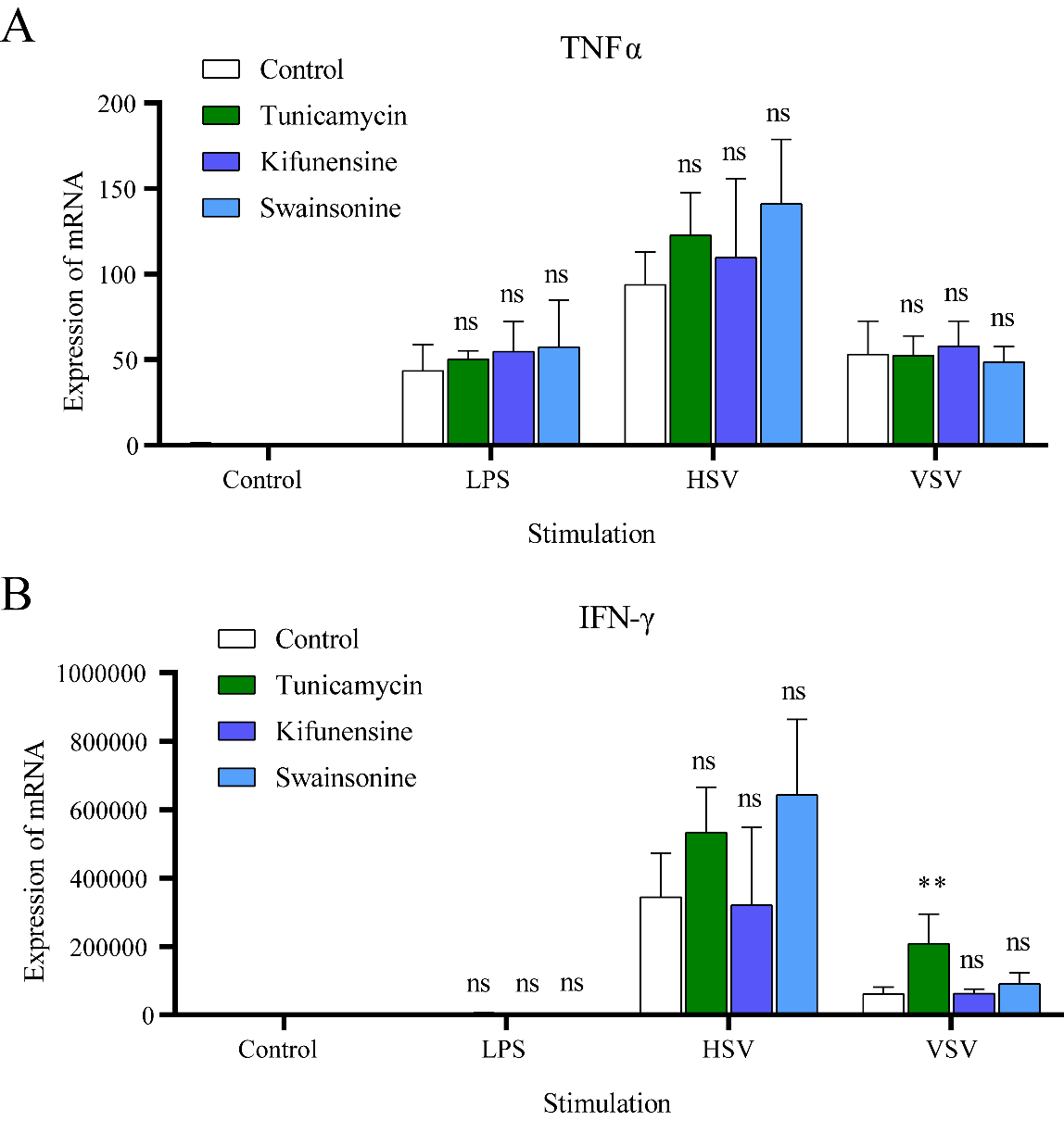


Figure S8. The mRNA expression of TNFα (A) and IFN-γ (B) in RAW264.7 cells under the treatment of the tunicamycin, kifunensine and swainsonine.

1. **Supporting tables (attached in additional tables)**
2. Table S1: iTraq labeled quantification of proteome
3. Table S2: Label free quantification of the N-glycoproteome
4. Table S3: Gene-concept network of significant GO terms enriched and their associations with top-related glycoproteins
